# Reference gene selection for RT-qPCR normalization in the drone testis of honey bee (*Apis cerana*) during meiosis stages

**DOI:** 10.1371/journal.pone.0347110

**Published:** 2026-04-09

**Authors:** Lifenfang Tao, Xinran Fu, Chunxiu Pang, Yanan Zhu, Jiaxing Huang

**Affiliations:** 1 State Key Laboratory of Resource Insects, Key Laboratory for Insect-Pollinator Biology of the Ministry of Agriculture and Rural Affairs, Institute of Apicultural Research, Chinese Academy of Agricultural Sciences, Beijing, China; 2 Guangxi Academy of Agricultural Sciences, Nanning, China; Guangzhou University, CHINA

## Abstract

Drones are the males in the honey bee population that provide high quantity and quality sperm to mate with the queen and ensure colony reproduction. To understand spermatogenesis in the drone testis at the molecular level, reverse transcription quantitative PCR (RT-qPCR) is a standard and useful means to investigate the target genes. However, a lack of appropriate reference genes hinders the accurate normalization of target genes. To identify stable reference genes in the drone testis during the meiosis stages of honey bee, we assessed eight candidate genes using BestKeeper, delta-CT, GeNorm, NormFinder, and RefFinder tools. Moreover, the expression pattern of *stat92e* and *dicer1* was used to validate the stability of the selected reference genes. The results showed that the most stable reference gene in the testis during meiosis stages varied according to different algorithms, and *gapdh* was the most stable reference gene in the drone testis during meiosis stages when integrating these different algorithms. In addition, *gapdh*, *rps18*, and the combination of *gapdh* & *rps18* were optimal reference genes to normalize the expression of the target genes in testis during meiosis stages, as validated by the expression of *stat92e* and *dicer1*. Our findings provide a conducive foundation for the accurate quantification of gene expression levels in the testis during the drone meiosis stages of the honey bee.

## Introduction

Honey bees are essential pollinators in natural and agricultural ecosystems, playing a vital role in maintaining ecological balance and supporting agricultural economies [1,2]. A healthy honey bee colony typically consists of a queen, thousands of workers and drones, exhibiting a strict division of labor within the colony. Drones are a vital component of the honey bee colony, and their primary function is to produce quantities of sperm and mate with queens effectively [3–5]. This means drones play an indispensable role in the process of population reproduction. Hence, research on drones mostly focuses on the reproductive system [4,6–12], especially on the testis, which are the organs responsible for spermatogenesis and sperm maturation [10]. The meiosis process is a key step in spermatogenesis, occurring in the testis during larval, pupal, and pharate adult stages before emergence [13]. Therefore, it is important to explore the molecular regulatory mechanisms involved in the meiosis process at these stages.

The real-time quantitative polymerase chain reaction (RT-qPCR) is a powerful molecular technique for quantifying the expression of specific genes under various environmental conditions and during tissue development [14]. To date, there is no suitable reference gene for the drone testis of the honeybee during the meiosis stages, which significantly limits research on the specific function of genes involved in spermatogenesis and the molecular regulatory mechanisms of spermatogenesis. Therefore, evaluating the stability of candidate reference genes in honey bee during the drone meiosis stages is necessary.

Stable reference genes play an irreplaceable role in the reliability and accuracy of RT-qPCR analysis. Typically, the reference genes used in honey bee drones for RT-qPCR are the common housekeeping genes, including *40S ribosomal protein S18* (*rps18*) [15], *60S ribosomal protein l32* (*rpl32*) [16], *elongation factor 1 alpha* (*ef1-α*), *glyceraldehyde 3-phosphate dehydrogenase* (*gapdh*), *β-actin related protein 1* (*β-actin*), *ribosomal protein 49* (*rp49*), *tubulin alpha-1 chain* (*tubulin*) [17], and *ribosomal protein s5* (*rps5*) [18]. Therefore, it’s preferable to test the stability of these genes in drone testis of honey bee during the meiosis stages.

*Apis cerana* is the native honey bee in Asia that evolved through long-term adaptation to the Asian diverse natural ecological environment. In this study, eight candidate reference genes were selected to perform RT-qPCR assay in drone testis of *A. cerana* during the meiosis stages. Five algorithms were applied to evaluate the stability of candidate reference genes: Bestkeeper [19], GeNorm [20], NormFinder [21], delta-Ct [22,23], and RefFinder [24]. Two genes (*stat92e* and *dicer1*) are specifically expressed in male germ cells and participate in the regulation of sex-specific gene expression in male germ cells and spermatogenesis, respectively [25,26]. In addition, *stat92e* and *dicer1* are highly expressed in the honey bee testis, which may be involved in drone spermatogenesis [27]. Therefore, *stat92e* and *dicer1* were quantified to validate the chosen best-ranked reference genes. Our results will contribute to providing the reliable reference genes of RT-qPCR in gene expression studies of the testis during drone development in honey bee.

## Materials and methods

### Drone testis samples

The drone larvae, pupae, and pharate adults of *A. cerana* were collected from three different colonies equipped with sister queens in the Yunnan Cangyuan Experimental Station of the Institute of Apicultural Research (IAR), Chinese Academy of Agricultural Sciences (CAAS), Cangyuan County, Yunnan Province (23˚18′00′′˚N, 99˚7′12′′E, 1251.4 m elevation), and the testis was dissected in cold PBS buffer immediately. Due to the testis of larvae from the first day larvae to the fourth day larvae were very fragile and could not be integrally separated from the surrounding fat body, the testis samples were digested from the fifth day larval stage (L5). According to the developmental morphology, the L5 larval stage is divided into three distinct phases: the feeding phase (L5F), the spinning phase (L5S), and the prepupal phase (PP). Pupae were categorized based on the progression of eye color, ranging from the white stage (Pw) to the pink stage (Pp), and then to the dark stage (Pd). Then, the pharate-adult stages were followed, during which the body gradually darkened from a light melanized state (Pdl) to an intermediate melanized state (Pdm), and ultimately to a fully pigmented state (Pdd) [10]. The dissected testis samples of nine development stages were immediately placed in Nitrogen-free RNA preservation solution (Solarbio, China) and stored at −80℃ for RNA extraction. The ethics committee of the Institute of Apicultural Research (IAR) approved the experimental protocol (Approval No.: MFSDWLLSC-2024-07; approval date: 6 August 2024).

### Total RNA extraction and cDNA synthesis

The testes from one drone were treated as one biological replicate, five biological replicates per development stage were generated. Total RNA was extracted with Trizol reagent (Invitrogen, USA) according to the manufacturer’s instructions. The testes were homogenized in Trizol reagent, and chloroform was added to separate the homogenate into a transparent upper aqueous layer (containing RNA), an intermediate phase and a red lower organic layer (containing DNA and protein). RNA was precipitated from the water layer using isopropyl alcohol. The RNA purity and concentration were detected using NanoDrop (ThermoFisher Scientific, USA). A total of 2 μg of RNA was used to synthesize first-strand cDNA with a FastKing RT Kit (Tiangen, China) according to the manufacturer’s instructions.

### Reference gene selection and primer design

Eight candidate reference genes of *A. cerana* were selected for the following RT-qPCR analysis: *β-actin*, *ef1-α*, *gapdh*, *rpl32*, *rps5*, *rps18*, *tubulin*, and *rp49*. The sequence information of these genes was obtained from the NCBI database (https://www.ncbi.nlm.nih.gov). The primers of each gene were designed by Primer3, and all primers were synthesized by TianYiHuiYuan Biotech (China). The gene information and primer information for the eight candidate reference genes and two target genes were summarized in Table 1.

**Table 1:** Primers of the candidate reference genes and target genes used for RT-qPCR and their amplification efficiency value (E) and the correlation coefficient (R^2^) of the qPCR standard curve.

Gene Symbol	Gene name	Accession No.	Primer sequence (5’-3’)	Product length(bp)	E (%)	R^2^
***rpl32***	60S ribosomal protein l32	XM017056470.3	F: TTATTCGTCATCAGAGTGR: TTCCAATTCCTTAACATTATG	200	95	0.999
***rps18***	40S ribosomal protein S18	XM017067400.3	F: GGTCGTCGTTATGCTAATR: ATCAGGAATCTTGTATTGTCTA	135	96	0.992
***ef1-ɑ***	elongation factor 1 alpha	OK158012.1	F: CGATGTTAAGCGTGTGAGR: CATACCTGTGTCCAAGATTC	194	107	0.999
***β-actin***	actin related protein 1	XM017065464.3	F: GTGTGATGGTCGGTATGGR: GCGGAGTTCGTTGTAGAA	161	108	0.999
***gapdh***	glyceraldehyde 3-phosphate dehydrogenase	XM017062468.3	F: CTCAGGTTGTTGCCATTAR: TTGCCTCTCGTTCACTAA	165	111	0.996
***tubulin***	tubulin alpha-1 chain	XM062086192.1	F: CGAACAAGCATATAACAAR: TGGAAGATAACGAAGATAA	156	93	0.981
***rp49***	ribosomal protein 49	XM062081709.1	F: TTGATAATAGAGTTCGTAGGR: CTGCTCACCTTAACATTA	193	161	0.992
***rps5***	ribosomal protein s5	XM017057553.3	F: TTCTTGTATCTGCTATCATCAR: GCCTGTCTCCTAACTGTA	82	228	0.873
***dicer1***	ribonuclease III	XM028669944.2	F: TATTGCTGTAATGCTCATAAR: ATTCCTATATCTCCACTAAGT	174	109	0.991
***stat92e***	signal transducer and transcription activator Stat92E	XM017062041.3	F: TATCATCAAGCAACAAGACAAR: CACCTCCTCCAACACTAA	148	108	0.996

### RT-qPCR analysis

The RT-qPCR was performed on Agilent Mx3000P. The RT-qPCR reactions were performed in a final volume of 10 μL containing 5 μL 2 × Talent qPCR Premix (Tiangen, China), 0.25 μL of 10 μM forward primers and 0.25 μL of 10 μM reverse primers, 4 μL cDNA template, and 0.5 μL double-distilled H_2_O. The reactions for reference gene selection were performed in triplicate, and for the validation of the selected reference genes were performed in duplicate, all reactions were performed under the following three-step cycling conditions: 95℃ for 2 min 20 s, 40 cycles of 95℃ for 5 s, 55℃ for 10 s, and 72℃ for 15 s, 1 melting cycle of 95℃ for 1 min, 55℃ for 30 s, and 95℃ for 30 s. The amplification specificity of the primers was determined by melting curves of qRCR reaction and by visualization on 1% agarose gel. Then, the PCR amplicons were separately cloned into a pGEM-T easy vector (Promega, USA), and the recombinant vector was transformed into *Trans1*-T1 phage-resistant, chemically competent cells (TransGen, China). The purified plasmid containing positive inserts was sequenced with the M13 forward universal primer by TianYiHuiYuan Biotech (China). Then the accuracy of the sequence of amplicon was confirmed by sequencing analysis. To further verify the high efficiency of the selected primer pairs, the amplification efficiency value (E) and the correlation coefficient (R^2^) of the RT-qPCR assays were calculated according to the standard curves assay.

### Data analysis

The cycle threshold (Ct) values were collected for the following data analysis. The gene-expression stability of the candidate reference genes was evaluated based on five statistical algorithms: BestKeeper [19], GeNorm [20], NormFinder [21], delta-Ct method [22,23], and the online platform RefFinder (https://blooge.cn/RefFinder/) [24]. BestKeeper is an Excel-based tool that identifies the most stable reference genes from a pool of candidate reference genes by analyzing the standard deviation (SD) and coefficient of variation (CV) of different reference genes. Lower SD and CV values suggest more consistent and stable expression of reference genes [19]. Likewise, the delta-Ct method determines the stability of reference genes by evaluating their average SD, and a lower SD value indicates a higher degree of stability for the reference gene [23]. The GeNorm algorithm assesses the stability of reference genes through M-value of reference genes. M-value is derived from the average pairwise variation of a gene compared to all other candidate genes, a lower M-value indicates more stable expression across the samples [20]. In addition, to ascertain the optimal number of reference genes for normalization, GeNorm computes the pairwise variation (V) between sequential normalization factors (V_n_ and V_n+1_). The value of V_n_/V_n+1_ below a threshold (commonly 0.15) implies that incorporating an extra gene offers negligible improvement in normalization, thereby indicating that the existing set of genes is adequate [20]. NormFinder uses a comprehensive model-based approach to evaluate the stability of reference genes by considering both intra- and inter-group variations, providing a robust method for selecting the most stable genes for accurate normalization in RT-qPCR studies. A lower stability value indicates higher stability, indicating that gene expression is less affected by experimental variations [21]. RefFinder provides a robust and comprehensive approach to assessing the stability of reference genes by integrating the strengths of multiple algorithms. By combining the results from GeNorm, NormFinder, BestKeeper, and delta-Ct method, RefFinder offers a more reliable and objective evaluation of gene stability, aiding researchers in selecting the most appropriate reference genes [24].

### Validation of reference genes

*Stat92e* and *dicer1* that might be involved in testis development of *A. cerana* were used as the target genes to validate the selected reference genes during drone development. The relative expression of *stat92e* and *dicer1* was normalized with a single reference gene or multiple reference genes using the 2^-△△Ct^ method. One-way ANOVA followed by Tukey’s post hoc tests was used for multigroup comparisons. All statistical analyses were performed by Microsoft Excel 2016.

## Results

### Amplification specificity and efficiency

**Fig 1:**
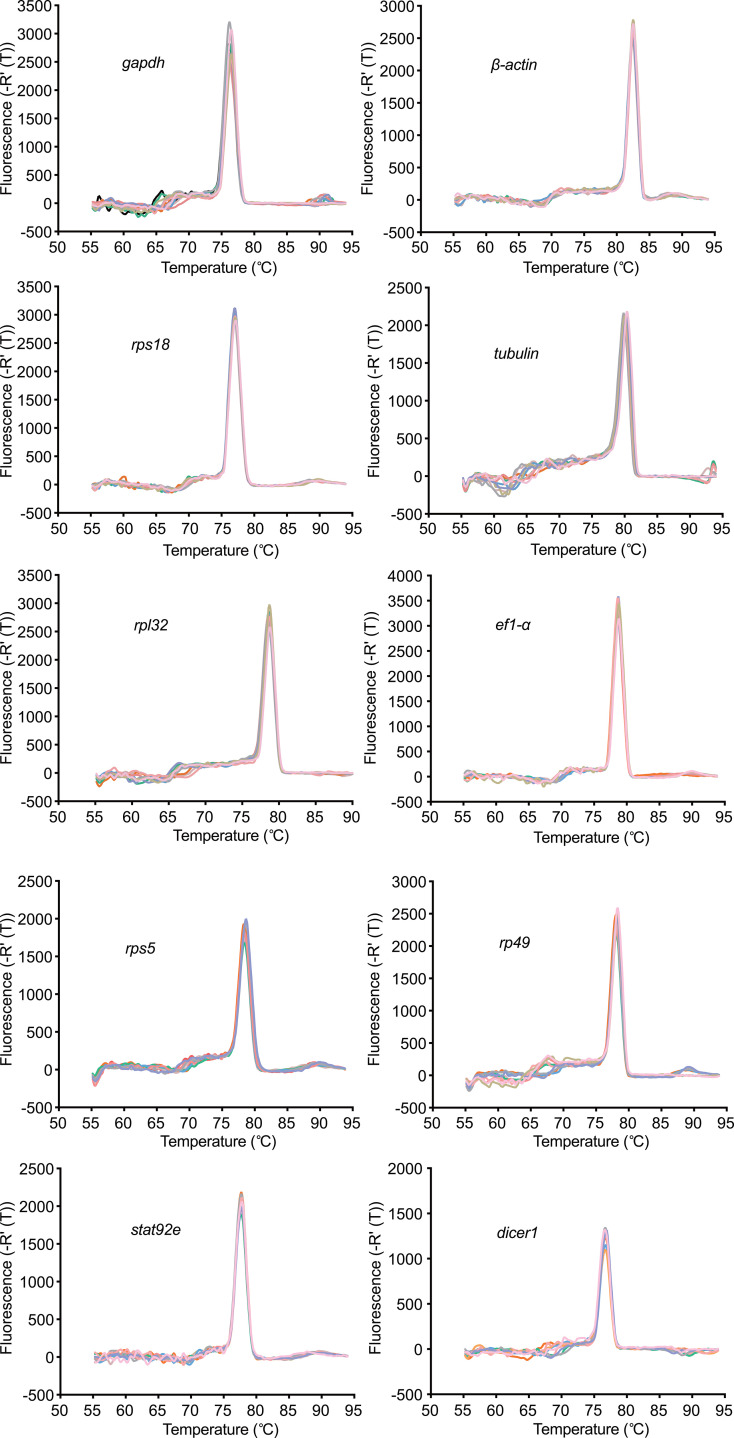
Melting curve analysis of the candidate reference genes and target genes. X-axis indicates the temperature (℃), y-axis indicates the fluorescence density. Melting curve analysis revealed a single and sharp peak, confirming the amplification of a specific PCR product.

To investigate the amplification specificity and efficiency of the primers for the eight reference genes and two validation genes, RT-qPCR assay was performed in drone testis of *A. cerana*. The RT-qPCR assay results showed a single peak in real-time melting curves of each gene (Fig 1). Then the nucleic acid gel electrophoresis assay of all amplicons was performed, and the results showed that a single band was displayed on the 1% agarose gel (S1 Fig). The amplification specificity of the selected primer pairs for these genes was confirmed by the single band and melting curves. In addition, the standard curves assay results showed that the R^2^ values of most reference genes were 0.991–0.999, and the amplification efficiency (E-value) of most reference genes ranged from 93% to 111%, except *rp49* and *rps5*, whose E-values reached 161% and 228% (Table 1). These results showed that the primers for the eight genes, except for *rp49* and *rps5,* had high specificity and amplification efficiency, making them suitable for further analysis.

### Expression profiles of reference genes during meiosis stages of drone

**Fig 2:**
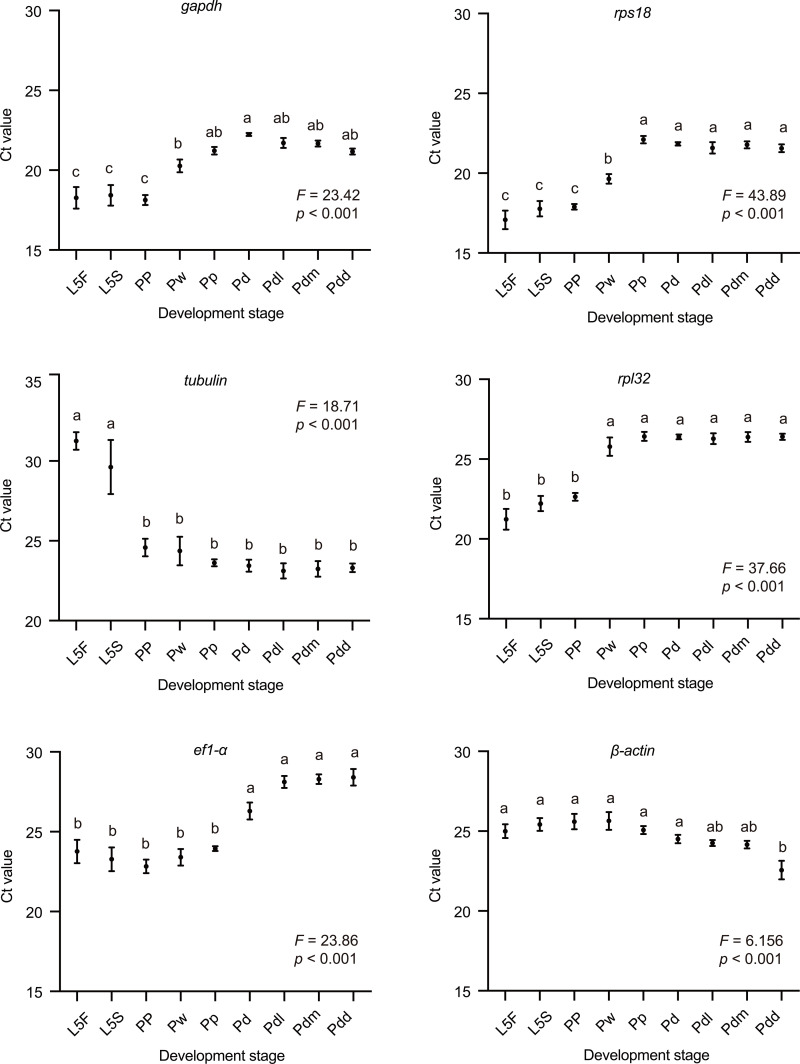
Cycle threshold (Ct) values of the six reference genes during meiosis stages of drone testis. X-axis represents the different development stages of drone, y-axis represents the cycle threshold. L5F: the fifth day larval stage of feeding phase; L5S: the fifth day larval stage of spinning phase; PP: the prepupal phase; Pw: white-eyed pupae; Pp: pink-eyed pupae; Pd: dark-eyed pupae; Pdl: light-body pharate adult stage; Pdm: intermediate-body pharate adult stage; Pdd: fully pigmented-body pharate adult stage. *n* = 5 biologically independent drones. Statistically significant differences are shown by different letters using one-way ANOVA followed Tukey’s multiple comparisons test. The data are shown as the mean ± SEM.

RT-qPCR assay was performed to verify the expression levels of the six candidate reference genes in the testis during the meiosis stages of the drone (Fig 2). The expression pattern of Ct values for the six reference genes showed significant variations irrespective of drone development stages (*p* < 0.001, *n* = 5, One-Way ANOVA), with the average Ct values of all samples ranging from 15.8 to 33.4. In addition, the Ct values showed that *gapdh*, *rps18*, and *rpl32* exhibited similar expression trends at different meiosis stages: lower expression levels in L5 stages and the highest expression levels in pupae and pharate adult stages. The results also indicated that *β-actin* had the smallest variation, followed by *gapdh*, *rps18*, and *rpl32*, while *ef1-α* and *tubulin* had the largest variation. Therefore, *β-actin*, *gapdh*, *rps18*, and *rpl32* were considered as relatively stable reference genes compared with other reference genes.

### Stability of the candidate reference genes during meiosis stages of the drone

**Fig 3:**
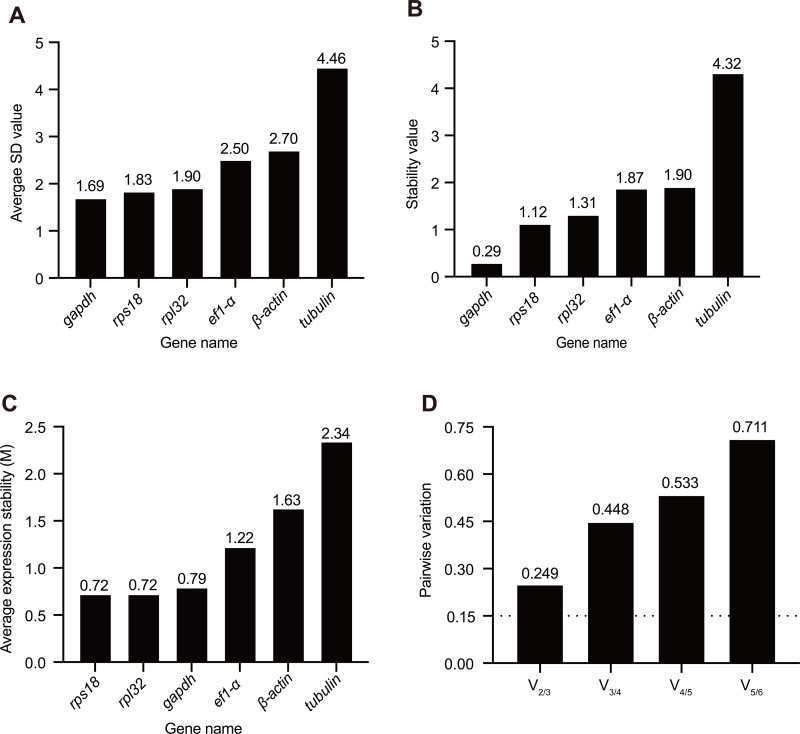
Robustness of candidate genes by delta-Ct analysis (A), NormFinder analysis (B), GeNorm analysis (C), and the pairwise variation values for the reference genes with GeNorm analysis (D). **(A)** Delta-Ct method determines the stability of reference genes by evaluating the average standard deviation (SD). A lower SD value indicates a higher degree of stability for the reference gene. **(B)** NormFinder method evaluates the stability of reference genes by considering both intra- and inter-group variations. A lower stability value indicates higher stability. **(C)** M-value is derived from the average pairwise variation of a gene compared to all other candidate genes, a lower M-value indicates more stable expression of the candidate genes across the samples. **(D)** GeNorm computes the pairwise variation (V) between sequential normalization factors (V_n_ and V_n+1_). The threshold (commonly 0.15) is used to assess the optimal number of reference genes. The pairwise variation of V_n_/V_n+1_ < 0.15 means n is the optimal number of reference genes selected for RT qPCR pairwise variation.

**Fig 4:**
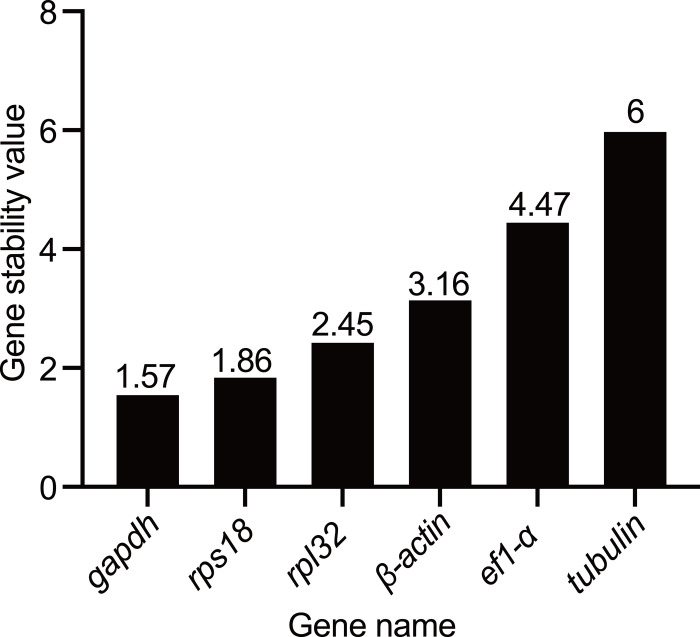
Comprehensive ranking of stability with RefFinder analysis. The stability of reference genes is analyzed by RefFinder through integrating the strengths of multiple algorithms. A lower gene stability value indicates the more stable expression of the candidate genes.

Five statistical algorithms were used for analyzing the stability of the candidate reference genes. Based on delta-Ct method and NormFinder analysis, *gapdh* was the most stable gene in testis among the meiosis stages of *A. cerana* drone with the stability value of 1.69 and 0.29, respectively, whereas *tubulin* was the least stable reference gene with the value of 4.46 and 4.32, respectively (Figs 3A and 3B). The stability of reference gene expression was evaluated by calculating the average M values through the GeNorm algorithm. During the meiosis stages of *A. cerana* drone, *rpl32* and *rps18* were the most stably expressed genes, with the lowest M value of 0.72, while *tubulin* was the least stably expressed gene, with the highest M value of 2.34 (Fig 3C). Furthermore, the results of the pairwise variation (V_n_/V_n+1_) during meiosis stages of drone calculated by GeNorm showed that all the pairwise variation values were above 0.15 (Fig 3D). As shown in Table 2, *β-actin* was identified as the most stable reference gene in *A. cerana* testis during meiosis stages of drone based on the Bestkeeper (CV ± SD = 3.62 ± 0.89), and the second and third stable reference genes were *gapdh* (CV ± SD = 7.28 ± 1.48) and *rps18* (CV ± SD = 9.10 ± 1.83), respectively. At the same time, the most unstable reference gene was *tubulin* with the highest CV and SD values (CV ± SD = 9.93 ± 2.5). The RefFinder comprehensive ranking of the reference genes showed that *gapdh* and *rps18* were the two most stable reference genes in the testis under the different meiosis stages of the *A. cerana* drone with the lowest stability values (Fig 4). Collectively, *gapdh* and *rps18* can be used further for normalizing the expression of target genes at the whole meiosis stages of the drone.

**Table 2:** Expression stability values of reference genes calculated by BestKeeper analysis.

Rank	Gene	SD	CV
**1**	*β-actin*	0.89	3.62
**2**	*gapdh*	1.48	7.28
**3**	*rps18*	1.83	9.10
**4**	*rpl32*	1.92	7.71
**5**	*ef1-α*	2.17	8.56
**6**	*tubulin*	2.50	9.93

### Validation of the selected reference genes

**Fig 5:**
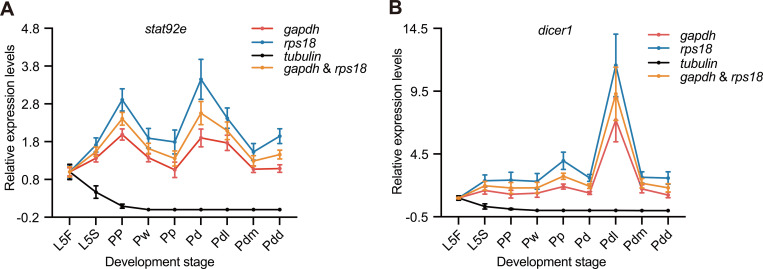
Relative expression levels of the target genes normalized with reference genes. X-axis represents the different development stages of drone, y-axis represents the expression level of the *stat92e* **(A)** and *dicer1* **(B)**. L5F: the fifth day larval stage of feeding phase; L5S: the fifth day larval stage of spinning phase; PP: the prepupal phase; Pw: white-eyed pupae; Pp: pink-eyed pupae; Pd: dark-eyed pupae; Pdl: light-body pharate adult stage; Pdm: intermediate-body pharate adult stage; Pdd: fully pigmented-body pharate adult stage. *n* = 5 biologically independent drones. The data are shown as the mean ± SEM.

To validate the stability of the expression of target genes, two relatively stable reference genes (*gapdh* and *rps18*) and the least stable gene (*tubulin*) were used to normalize gene expression of *stat92e* and *dicer1*. The results showed that the expression of *stat92e* in testis displayed higher levels at stage PP and Pd normalized with *gapdh* and *rps18* alone and with the combination of the two reference genes, while the expression of *stat92e* normalized with *tubulin* displayed the continuous declined tendency, showing the significantly reduced expression levels during the whole meiosis stages of drone (Fig 5A and S3A Fig). In addition, the expression of *dicer1* in testis normalized with *gapdh*, *rps18*, and their combination during the drone development showed the highest levels at Pdl stage and the lower expression levels at other stages, while normalized with *tubulin*, the expression level of *dicer1* showed significantly lower expression levels during the meiosis stages of drone (Fig 5B and S3B Fig).

## Discussion

The primary function of the drone is to provide high-quality sperm for offsprings genetics, therefore the research on reproductive system is indispensable. To investigate the reproductive development of drone bees at the molecular level, an appropriate and stable reference gene is necessary for accurate RT-qPCR during all drone developmental stages, even if the reference genes exhibit variability in different developmental stages [28,29]. To date, there is no research on selecting internal reference genes for the male reproductive system during the meiosis process of *A. cerana* drones. In the current study, we investigate the expression levels of eight candidate reference genes (*β-actin*, *ef1-α*, *gapdh*, *rpl32*, *rps5, rps18*, *tubulin*, *and rp49*), and evaluate their expression stability in testis during drone development using five different algorithms (Bestkeeper, GeNorm, NormFinder, delta-Ct, and RefFinder). According to the analysis of algorithms and validation of target genes, our results showed that *gapdh*, *rps18*, and their combination was sufficient as an internal reference gene in the testis during the meiosis stages of the drone.

Optimal primers are the basis for amplifying target genes, and an amplification efficiency value of primers between 90% and 110% is considered effective [30]. In this study, the melting curve and amplicon of the two primer pairs of *rp49* and *rps5* were single and specific (Fig 1 and S1 Fig). In contrast, the amplification efficiency values of primers for *rp49* and *rps5* were 161% and 228%, respectively (Table 1), which exceeded 110%. Besides, the Ct values of *rp49* and *rps5* during drone development were over 30 and 35 (S2 Fig), indicating the lower expression levels of the two genes in the testis. Higher Ct values might exceed the threshold when the templates were diluted for the amplification efficiency assay, making *rp49* and *rps5* unsuitable as candidate genes at different meiosis stages. Therefore, *rp49* and *rps5* were excluded from the subsequent analysis, and the other six candidate reference genes (*β-actin*, *ef1-α*, *gapdh*, *rps18*, *tubulin*, and *rpl32*) were suitable for further analysis.

Typically, one reference gene is sufficient for RT-qPCR to determine the expression of a target gene [31,32]. However, in recent years, more and more studies show that the combination of two and more reference genes is necessary for analyzing the target genes in RT-qPCR assay [29,33]. GeNorm algorithm was also used to determine the optimal number of reference genes for TR-qPCR standardization by calculating the pairwise variation value (V_n_/V_n+1_) [20]. The pairwise variation value below the cutoff point of 0.15 indicates that the additional reference gene is not necessary [20]. Our results showed that all the V_n_/V_n+1_ values are above 0.15 (Fig 3D). The optimal number of reference genes was not determined in the testis during the meiosis stages of the drone, and using a single reference gene might be appropriate. However, 0.15 is just a theoretical value, and higher cutoff values of V_n_/V_n+1_ have been used elsewhere [34]. Our results also showed that, respectively, using *gapdh*, *rps18*, and the combination of *gapdh* and *rps18* as reference genes, the expression of *stat92*e and *dicer1* displayed the same expression trend during the meiosis stages of the drone (Fig 5). *gapdh* and *rps18* have been suggested to be the optimal reference genes to determine the seasonal or labor-specific gene expression in the various tissues (head, thorax, and abdomen) of *A. mellifera* [33]. Furthermore, *gapdh* and *rps18* are suitable reference genes in the *A. mellifera* head after *Escherichia coli* infection [32,35]. Consistent with previous research, *gapdh*, *rps18*, or their combination can be used in RT-qPCR assays under various meiosis stages, and experimental conditions in different tissues.

The tested candidate reference genes are used to assess the expression of odorant binding proteins and activity rhythm-dependent genes in the honey bee drone antenna [15,17], and the expression of a body coloration-related gene in drone bees [16]. All these reference genes are used individually to calculate the expression of target genes at a particular stage or tissue, whereas our study verified the stability and accuracy of these candidate reference genes during meiosis stages of drone testis. Furthermore, all candidate reference genes have been confirmed to be the most suitable reference genes under different species, physiological states and environmental stress [32,36–41]. For example, *rpl32* exhibited exceptional expression stability in different tissues of *Tribolium castaneum* larvae following *Staphylococcus aureus* infections [37]. In *Paederus fuscipes*, *ef1-α*, *tubulin*, *rps18*, and *β-actin* varied significantly depending on developmental stage, tissue, diet, and temperature conditions [40]. Therefore, the selection of the most suitable reference genes for RT-qPCR requires comprehensive consideration of multiple factors, including the species, physiological state, and experimental stress. Consistent with this conclusion, all the candidate reference genes displayed fluctuating Ct values in the drone testis during the meiosis stages, suggesting that the housekeeping genes can be affected by physiological differences among the larval, pupal, and pharate adult stages. In accordance with our results, reference genes display fluctuating expression during the embryonic and postembryonic development of honey bees [29]. Additionally, the stability of the reference gene was verified during drone development, from larvae to the pharate adult. At the same time, the reproductive system will develop to the mature stage after the honey bee emerges. The physiological status of other tissues in the drone reproductive system, such as the seminal vesicle and mucous gland, changes from larvae to adults [42]. Thus, further studies are needed to explore the suitable reference genes in different reproductive tissues and during different developmental stages.

## In conclusion

This study analyzed eight commonly used reference genes for RT-qPCR assays in the testis of *A. cerana* drones using five algorithms during the meiosis stages. The study selected *gapdh*, *rps18*, and their combination as the optimal reference genes to verify the expression of target genes by RT-qPCR. The findings will contribute to further research focusing on testis development and spermatogenesis in the drone bee.

## Supporting information

S1 Fig PCR amplification of the ten candidate genes from the testis of *A. cerana* drone. Each amplicon was visualized on 1% agarose gel.
(EPS)

S2 Fig Cycle threshold (Ct) values of the *rps5* and *rp49* during meiosis stages of drone testis. X-axis represents the different development stages of drone, y-axis represents the cycle threshold. L5F: the fifth day larval stage of feeding phase; L5S: the fifth day larval stage of spinning phase; PP: the prepupal phase; Pw: white-eyed pupae; Pp: pink-eyed pupae; Pd: dark-eyed pupae; Pdl: light-body pharate adult stage; Pdm: intermediate-body pharate adult stage; Pdd: fully pigmented-body pharate adult stage. *n* = 5 biologically independent drones. Statistically significant differences are shown by different letters using one-way ANOVA followed Tukey’s multiple comparisons test. The data are shown as the mean ± SEM.
(EPS)

S3 Fig Relative expression levels of the target genes normalized with reference genes. Relative expression levels of the target genes *stat92e* **(A)** and *dicer1* **(B)**, normalized with a single reference gene (*gapdh*, *rps18*, *tubulin*) and a combination of two paired reference genes (*gapdh* & *rps18*) during the meiosis stages of drones. X-axis represents the different development stages of drone, y-axis represents the expression level of the *stat92e* and *dicer1*. L5F: the fifth day larval stage of feeding phase; L5S: the fifth day larval stage of spinning phase; PP: the prepupal phase; Pw: white-eyed pupae; Pp: pink-eyed pupae; Pd: dark-eyed pupae; Pdl: light-body pharate adult stage; Pdm: intermediate-body pharate adult stage; Pdd: fully pigmented-body pharate adult stage. *n* = 5 biologically independent drones. Statistically significant differences within the same development stage are shown by different letters using one-way ANOVA followed Tukey’s multiple comparisons test. The data are shown as the mean ± SEM.
(EPS)
